# The Near-Miss Effect in Slot Machines: A Review and Experimental Analysis Over Half a Century Later

**DOI:** 10.1007/s10899-019-09891-8

**Published:** 2019-09-14

**Authors:** Jeffrey M. Pisklak, Joshua J. H. Yong, Marcia L. Spetch

**Affiliations:** Psychology, P217 Biological Sciences Building, University of Alberta, Edmonton, AB T6G 2E9 Canada

**Keywords:** Gambling, Reinforcement, Near-miss, Near-hit, Pigeons, Humans

## Abstract

In games of chance, a near miss is said to occur when feedback for a loss approximates a win. For instance, obtaining “cherry–cherry–lemon” on a slot machine could be considered a near miss. Sixty-six years ago, B.F. Skinner first proposed the idea that near-miss events might reinforce continued play in slot machines, and despite some inconsistencies in the experimental literature, belief in this “near-miss effect” has remained strong. In the present manuscript, we will review this literature and present experimental assessments of the near-miss effect on the frequency of the gambling response. Experiment 1 used a tightly controlled resistance-to-extinction procedure in pigeons to evaluate the putative reinforcing effect of near misses relative to a control “far-miss” reel pattern. Experiment 2 extended Experiment 1’s procedure to human participants. The results of both experiments failed to support the near-miss effect hypothesis. Experiment 3 used a further simplified procedure to assess the validity of the resistance-to-extinction paradigm when a probable conditional reinforcer was present on the reel stimuli. Although a clear conditional response was obtained from the reel, subsequent testing in extinction revealed no conditionally reinforcing function of this stimulus on operant response frequency.

## Introduction

Near misses, also called near hits or near wins, are said to occur when the elements of a game or task “suggest” to a player that they have almost achieved a favourable result. A good example is provided by Witts et al. (2015): consider a novice player making repeated free throws in basketball. With each successive throw, variations in the throwing technique that get the ball closer towards the hoop increase in probability (i.e., are selected for). This is an instance where the “near miss” has a clear reinforcing function on the player’s free-throw behaviour.

Near misses also occur in games of chance, but the crucial difference is that in a game of chance the outcome is a random event. On a standard slot machine, if a win is signalled by “cherry–cherry–cherry,” then “cherry–cherry–lemon” would be considered a near miss. Modern slot machines contain a pseudo-random number generator (RNG) that cycles through about 4.3 billion distinct values continuously at approximately 1000 values per second. For each bet, the machine selects the cycle’s current position and outputs the correlated reel positions onto the display (Schüll 2012). Unlike the free throw in basketball, no amount of practice will improve the odds of winning at the slot machine. Moreover, receiving a near miss is no more informative about an upcoming win than any other type of miss. This raises an important question: if the near miss inside a game of chance is independent of a win and cannot be used to increase the chance of a win, then why is it considered a “near” miss?

The answer seems to be that the near miss is visually “nearly” a win. For example, cherry–cherry–lemon looks more similar than cherry–lemon–lemon to a win signalled by three cherries. In the case of basketball free throws, visual aspects of the situation provide helpful feedback to the player. Near-miss feedback on slot machines, however, offers no practical use for improving performance. One possibility is that the visual aspect of the near miss is exploiting learning processes—most notably conditional reinforcement—that evolved to detect contingent (i.e., non-random) outcomes. In this manuscript, we will first review the existing literature pertaining to the reinforcing effect of near misses on gambling persistence, and then we will present the results of experiments specifically designed to test possible reinforcing effects of near-miss stimuli on gambling persistence in both humans and pigeons.

## Conditional Reinforcement

Conditional reinforcement is thought to play a significant role in gambling behaviour. For example, audio-visual stimuli correlated with winning on slot machines may acquire conditionally reinforcing properties that encourage further play (Cherkasova et al. 2018). As early as 1953, B.F. Skinner discussed conditional reinforcement in what is likely the first scientific account of near misses. Skinner’s (1953) account drew upon conditional reinforcement as one of the plausible methods casinos were using to exploit their patrons and it is still cited as an explanation of the *near*-*miss effect*—the belief that near-miss events contribute to an increased frequency of gambling. More formally, we define the near-miss effect as a reinforcing function of near-miss events on total frequency of play in games of chance. Skinner’s original hypothesis rested on two critical factors: (1) that conditional reinforcement is based on pairing (i.e., contiguity), and (2) that near misses do in fact increase the frequency of betting responses. When Skinner proposed this, the available evidence largely supported a pairing account of conditional reinforcement, and the reinforcing effect of near misses was a sensible a priori prediction. Subsequent research, however, has shown that the pairing account is inadequate since it ignores nuances that influence and characterize this type of learning (Lattal 2013; Rescorla 1988), and because pairing is not sufficient to produce a conditionally reinforcing effect (e.g., Schoenfeld et al. 1950; Stubbs 1971). Contemporary accounts of conditional reinforcement now emphasize contingency, with two prominent mathematical models being *delay reduction theory* and the *hyperbolic decay model* (Fantino 1977; Mazur 1997). Despite wide acceptance of these models in behaviour analysis, the near-miss effect continues to be conceptualized through the pairing formulation (e.g., Kassinove and Schare 2001).

Experimental tests of conditional reinforcement often employ chained schedules in which two or more schedules of reinforcement, signalled by unique exteroceptive stimuli, are presented successively. Conditionally reinforcing effects of a stimulus within the chain can then be assessed by instituting extinction and comparing responding in the presence and absence of the putative conditional reinforcer. As noted by Kelleher and Gollub (1962), extinction procedures avoid the confounding effects of unconditional reinforcers in testing but can inadvertently introduce other problems. For example, extinction may alter the context such that stimuli function differently than in training (i.e., are “viewed” differently by the organism). Kelleher and Gollub also noted that extinction often produces only small effects—presumably because the conditional reinforcer is being simultaneously tested and extinguished. Finally, a problem specific to near misses is that conventional chained procedures that successfully produce conditional reinforcement have a logical predictability (i.e., contingency) between the putative conditional reinforcer and subsequent unconditional reinforcer. The classic slot machine, however, provides random outcomes with no contingency between the occurrence of near-miss stimuli and the subsequent occurrence of a winning outcome. It is not clear, therefore, why near-miss stimuli should be assumed to have conditionally reinforcing effects.

Furthermore, near misses can be conceptualized globally (i.e., cherry–cherry–lemon could be viewed as a single stimulus) or locally (i.e., each element as a separate stimulus). From a global view, the conditionally reinforcing effect of win-related stimuli may generalize better to near misses than to other, more dissimilar, misses (Belisle and Dixon 2016; Daly et al. 2014). Evidence consistent with a stimulus generalization account comes from findings that reel outcomes more visually similar to wins will generate longer latencies that are more like the latencies which occur after a win. However, it has been found that latencies are sometimes shorter—not longer—for stimuli that approximate a win (Dixon et al. 2013). Most problematic is that these studies have focussed only on latency and have not reported on overall rates of responding. Reinforcers increase response rates, leading to more cumulative responses across an interval of time despite post-reinforcement pausing (Ferster and Skinner 1957). Thus, evidence that the reinforcing function of winning stimuli generalizes to near misses also requires a demonstration that near misses increase the overall number or rate of bets. Near-miss events could also potentially function as conditional reinforcers by operating locally. For example, if cherry–cherry–cherry signals a win, then a cherry in the first reel position signals that the odds of winning have increased. However, the outcome of the spin is resolved quickly, so it is not clear that this brief change in probability signalled by the stimuli would be sufficient to conditionally reinforce gambling persistence.

## Animal Research

Despite calls for increased experimental analysis of gambling behaviour employing non-human animals (Weatherly and Phelps 2006), animal research on the near-miss effect is fairly sparse. This is surprising given the historical precedence of animal research on questions of reinforcement generally. Nevertheless, a few studies warrant discussion.

Using a refined slot-machine analogue (Peters et al. 2010; Weatherly and Derenne 2007), Winstanley et al. (2011) measured rates of extinction for two groups of rats’ responses on a *roll* lever. Alongside various neuropharmacological treatments, one group of eight rats received trials that contained near misses and another group of eight received no near-miss trials. The rats obtained food they won by responding on a *collect* lever. Pressing this lever on loss trials incurred a timeout penalty, thus making it advantageous to only press when winning cues were present. Rates of extinction on the *roll* lever were not significantly different between the two groups. Collect lever responses increased linearly as a function of win similarity and were more frequent after a near miss than, for instance, a full loss, which the researchers suggested may reflect “a process similar to a ‘near-miss’ effect” (p. 917). While this finding is consistent with a stimulus generalization account of near-misses, it should not be ignored that the critical extinction measure on the more relevant roll lever produced no such effect. Earlier research using similar methods likewise found no increase in persistence on the roll lever (Peters et al. 2010).

Other work has explored the reinforcing effect of near misses in choice paradigms. Two notable studies examined pigeons’ persistence on concurrently available alternatives (Fortes et al. 2017; Stagner et al. 2015). The pigeons consistently preferred alternatives containing no near misses across various manipulations. The findings are consistent with both delay reduction theory and the hyperbolic decay model’s interpretation of conditional reinforcement.

Another choice study analyzed matching (Davison and McCarthy 1988) between rates of responding and reinforcer value (Rice and Kyonka 2017). Results showed a consistent bias, across three pigeons, towards a “gambling” option containing near misses relative to a certain option that contained no losses (and therefore no near misses). However, this same bias was not observed when the gambling option containing near misses was tested against a similarly probabilistic option that contained no near misses.

## Human Research

Although there is considerable evidence that near-miss events can affect subjective measures (e.g., self-reports) and physiological responses (e.g., skin conductance, heart rate, or brain activity), there have been surprisingly few direct experimental tests of the presumed reinforcing function of near misses on gambling persistence. Effects on subjective motivation or physiological responses are consistent with a reinforcing effect on gambling frequency, but without a direct behavioral measure they do not actually demonstrate such an effect. For example, Dymond et al. (2014) found that increased activity in win-related brain regions was correlated with near misses (a similar effect was seen in pigeons’ neurophysiological responses to near misses; see Scarf et al. 2011) and with a trait measure of gambling propensity. They claimed to find “convincing evidence of a role for reward-related brain responses to near-miss outcomes, particularly in the insula, in maintaining PG [problem gambling]” (p. 216). Although the inference that near misses enhanced the activity in win-related brain regions is justified, the contribution of these near misses to problem gambling was based on a questionnaire with no direct assessment of the relationship between the near misses and the players’ actual gambling behaviour. Similar limitations exist in other studies (Billieux et al. 2012; Clark et al. 2009, 2013; Dixon et al. 2013; Habib and Dixon 2010).

A few experimental studies are frequently cited for providing evidence that near misses have a reinforcing function. Strickland and Grote (1967) found that significantly more participants who saw winning symbols occur more frequently on the earlier presented reels of a slot machine (i.e., more near misses than far misses), opted to continue playing than participants who saw more far misses than near misses. However, the average number of trials played by participants who opted to keep playing did not significantly differ between the two groups. Reid (1986) attempted two systematic replications of Strickland and Grote’s study. One used a card-based version of the slot machine task and the other used simulated slot machines. Only the latter showed the same pattern of results obtained by Strickland and Grote but neither replication obtained significant effects.

More recently, Kassinove and Schare (2001) manipulated the frequency of near misses on a four-reel slot machine simulation that participants played for money. Near misses had a 15%, 30%, or 45% chance of occurring for different groups. Participants were then put onto an extinction condition that removed both wins and near misses and could continue to play for as long as they desired. The 30% group showed the most persistence during extinction. A self-report administered after extinction showed no group differences on willingness to return to play in the future. The authors accounted for their findings in terms of conditional reinforcement, assuming that the 45% near miss frequency was presented too frequently (leading to respondent extinction), whereas the 15% condition was presented too infrequently (not paired enough). Across the 15%, 30%, and 45% groups, means (SD) of 5.88 (8.06), 10.26 (11.47), and 6.66 (8.22) responses in extinction were obtained respectively. This is one of the most frequently cited studies of evidence for a reinforcing function of near misses. However, it has some potential problems. First, Kassinove and Schare analyzed their extinction data using parametric statistics, which can be problematic for highly skewed data. In extinction, the probability of responding declines after each subsequent unreinforced response, making the data heavily bounded by zero. Additionally, if one assumes their data is symmetric then, based on their obtained variances, approximately 25% of their data points in the 30% group would fall below zero, which is not possible. Consequently, the obtained means and variances may not represent the population’s true value, particularly in the 30% condition which had substantially higher variance than the other groups, and thus a stronger relative pull of the mean under conditions of skewness. A similar criticism could potentially be made for other extinction- or persistence-based studies (Ghezzi et al. 2006; Reid 1986; Strickland and Grote 1967). A second concern is that extinction was tested without near misses, i.e., without the presence of the putative conditional reinforcer, which makes the observed difference puzzling. Finally, across three experiments, Ghezzi et al. (2006) attempted to replicate the findings of both Kassinove and Schare and Strickland and Grote by having participants play a simulated slot machine for points. Despite marginally larger group sample sizes than Kassinove and Schare, only one of their three experiments revealed a main effect of near-miss density on gambling persistence, and the most effective density was 66%. Overall, Ghezzi et al.’s findings were not commensurate with the original papers. Although there were notable procedural differences from the original two studies, this failure to replicate is concerning for the robustness of the near-miss effect on gambling persistence.

In another well-cited study, Cote et al. (2003) had participants play a three-reel video slot machine for money. The first phase contained 48 trials, programmed to give 12 near misses and 9 wins. In the second phase participants were, unknowingly, placed onto extinction. For an experimental group, extinction removed wins only. For a control group, extinction removed both wins and near misses. Participants could stop playing at any point and keep their earnings. Non-parametric analyses revealed that participants in the experimental group played significantly more during extinction than the control group. Although this study is cited as strong evidence of a near-miss effect, we believe there is a noteworthy concern that precludes it from providing evidence that near misses in a typical slot machine would increase gambling persistence. Specifically, in the first phase of the experiment, each win was preceded by a near miss. This contingency meant that each time a near miss was encountered, a win would be 75% certain to occur on the next trial, and thus near-misses actually predicted an increased chance of winning. This contingency would be expected to produce reinforcing effects, but it does not exist in typical slot machines.

An experiment by MacLin et al. (2007) gave recreational slot machine gamblers three concurrently available three-reel slot machines to play for points. Each machine had a specific frequency of near-miss presentations: 15%, 30%, or 45%. After 100 required trials, participants could choose to keep playing for a chance to earn a cash prize for the highest score among a pool of participants. However, at this point, the machines ceased to pay out wins (i.e., extinction). Participants persisted most with a 45% frequency and least with a 15% frequency on average, but the difference was not statistically significant.

Other studies that did not employ persistence-based methodologies have found similarly varied results ranging from support (Kurucz and Koermendi 2012; Tan et al. 2015) to lack of support (Witts et al. 2015) for a near-miss effect. Interestingly, Sundali et al. (2012) examined real casino data from electronic roulette terminals. They modelled the data of 36 players using regression analyses and found no evidence that near misses had reinforced the gamblers’ playing in terms of time spent playing or number of bets placed.

The variance across the aforementioned studies does not appear to be a function of the population sampled. Most did not specifically recruit problem gamblers and some used the South Oaks Gambling Screen (Lesieur and Blume 1987) to exclude probable pathological gamblers (Cote et al. 2003; Kassinove and Schare 2001; MacLin et al. 2007; Witts et al. 2015). Of these, only MacLin et al. (2007) sampled recreational gamblers. Strickland and Grote (1967) recruited rural high-school students and other studies recruited undergraduate students (Ghezzi et al. 2006; Kurucz and Koermendi 2012; Reid 1986; Tan et al. 2015).

The number of training trials ranged between 20 and 100 trials across studies. Of the studies showing increased persistence, Cote et al. (2003) used 48 trials, Kassinove and Schare (2001) used 50 trials, and Strickland and Grote (1967) used 100 trials. Interestingly, Ghezzi et al. (2006) varied the number of training trials (25, 50, 75, and 100) and found a significant effect on persistence only after 25 trials, with no trends as a function of training trials, and no interaction with near-miss density. This raises an important question: What is an appropriate amount of training in these studies? Generally, more training seems preferable to less training and this may be especially true for participants with less experience playing games of chance. Their expectations about gambling may conflict with the real-life gambling contingencies that experiments try to model, thus creating biases that may go unchallenged at low levels of training. Therefore, the results of novice gamblers may be different from more experienced gamblers, particularly with low levels of training.

Perhaps because of Skinner’s influence, the suggestion that near misses are functioning as conditional reinforcers has been largely taken for granted, yet our review of experimental studies has revealed inconsistent results and inconclusive evidence for an effect of near misses on gambling persistence. Many human studies have used either real lottery terminals or programs designed to mimic casino slot machines, which enhance external validity but can complicate the analyses of basic effects and comparisons between studies. Motivation levels can differ widely between individuals, and basic parameters such as the population sampled and the number of trials often vary across experiments. Arguably, the pursuit of external validity has hindered the establishment of internal validity of near-miss research. What seems required is a more controlled analysis reminiscent of the work Skinner favoured.

Pigeons have had a long and storied history in the study of conditional reinforcement and learning in general (Logue 2002). Many properties of pigeons make them suitable for a controlled analysis of near-miss effects: they have excellent visual acuity, easily regulated motivation levels, and exhibit steep rates of temporal discounting relative to other common laboratory animals (Stevens and Stephens 2009), which is pertinent for gambling research because problem gamblers have been found to discount delayed rewards more than non-gamblers (Dixon et al. 2003; Petry and Madden 2010; Reynolds 2006). Some have theorized that steep delay discounting, together with reinforcement schedules, is responsible for much of the maladaptive behaviour exhibited by problem gamblers (Rachlin et al. 2015). Enhanced experimental control can make behavioural data obtained through animal models more reliable than studying humans, although results should be verified in humans whenever feasible.

## Overview of Experiments

Experiments 1 and 2 used a resistance-to-extinction paradigm to test the reinforcing function of a near-miss stimulus pattern against a far-miss control pattern on both pigeons and humans. Using logic similar to the two notable experimental demonstrations of a near-miss effects (Cote et al. 2003; Kassinove and Schare 2001), if near-miss events function as conditional reinforcers more than other types of misses, then greater resistance to extinction should occur in conditions with a higher frequency of near-miss events than far-miss events.

Experiment 3 was designed to address the lack of a near-miss effect in Experiments 1 and 2. This experiment used pigeons and did not involve near-miss events. Instead, one of two single reel stimuli was contingently paired with food and its ability to elicit a conditional response was verified. The presence of that conditional stimulus was then tested during extinction to assess the validity of an extinction-based test of conditional reinforcement on behavioural persistence.

## Experiment 1a and 1b

### Methods

#### Subjects and Apparatus

For both Experiment 1a and 1b, homing pigeons (*Columba livia*) were randomly selected from a University of Alberta colony room for the experiment. A sample of eight pigeons was used, which is the same as employed in Stagner et al. (2015), and larger than other near-miss studies with pigeons (Fortes et al. 2017; Rice and Kyonka 2017; Scarf et al. 2011); the higher levels of experimental control in animal research and balanced within-subject design increases statistical power. Subjects were housed in 65 × 27 × 70 in. flight cages in a colony room maintained at 20 °C and a 12-h daylight cycle from 6:00 a.m. to 6:00 p.m. MST. All birds had free access to vitamin-enriched water and crushed oyster shell grit in the colony room. Subjects were maintained at 80% of their free-feeding weight by adjusting their post-experiment feeding of Mazuri Gamebird food pellets (PMI Nutrition International).

Six custom-built operant boxes were equipped with Carrol Touch infrared touchscreens (Elo Touch Systems, Inc., Menlo Park, CA) to detect pecking responses. Stimuli were presented on a centrally-mounted 17” Viewsonic LCD monitor located at the back wall of each chamber. Speakers in the operant boxes continuously played white noise to mask external sounds. The sound pressure levels were equalized in each operant box at 65 dB via A-weighting filter with a Brüel and Kjær Type 2239 Integrating Sound Level Meter. Two 2 × 2 in. feeding ports equipped with food hoppers, flanked the monitor. Access to food was controlled by Colbourne H20-94 photocell sensors that detected entry into the ports. Stimuli were presented and responses were logged using E-Prime^®^ 2.0 Professional software. A $${\raise0.5ex\hbox{$\scriptstyle 1$} \kern-0.1em/\kern-0.15em \lower0.25ex\hbox{$\scriptstyle 8$}}$$ in. thick white plastic barrier was mounted in front of the screen to prevent errant behaviours (e.g., subject’s wings, bodies, and feet contacting the screen) from interfering with the touch screen’s ability to record pecking responses. The barrier had four holes cut in it: three horizontal circles were cut to 1½ in. diameter near the top to allow for visual identification of the reel stimuli and a 1 in. diameter hole was cut beneath the middle of the three circles to allow for pecking responses.

Stimuli were presented on a black background and were aligned behind the holes cut in the barrier. A white pecking circle was presented at the smaller 1 in. hole. The win and miss patterns (i.e., reel patterns) were presented at the upper three circles. For Experiment 1a, each reel pattern consisted only of those elements comprising the winning reel pattern, which was displayed as three red circles. A *win* occurred when all three circles were red. A *near miss* occurred when only the left and middle circles were red. A *flanked miss* occurred when only the left and right circles were red. A *far miss* occurred when only the middle and right circles were red. Finally, a *single miss* occurred when only one circle was red. On single misses, each of the three locations had an equal chance of turning red. Non-illuminated circles were left black. Experiment 1b was identical but with the exception that the non-illuminated circles were coloured blue. For example, a trial displaying a far-miss would be presented as blue–red–red, with the blue signalling non-reinforcement. We denote red circles as putative S^D^ stimuli (i.e., stimuli that occasion reinforcement) and blue circles as putative S^Δ^ stimuli (i.e., stimuli that occasion nonreinforcement).

**Fig. 1:**
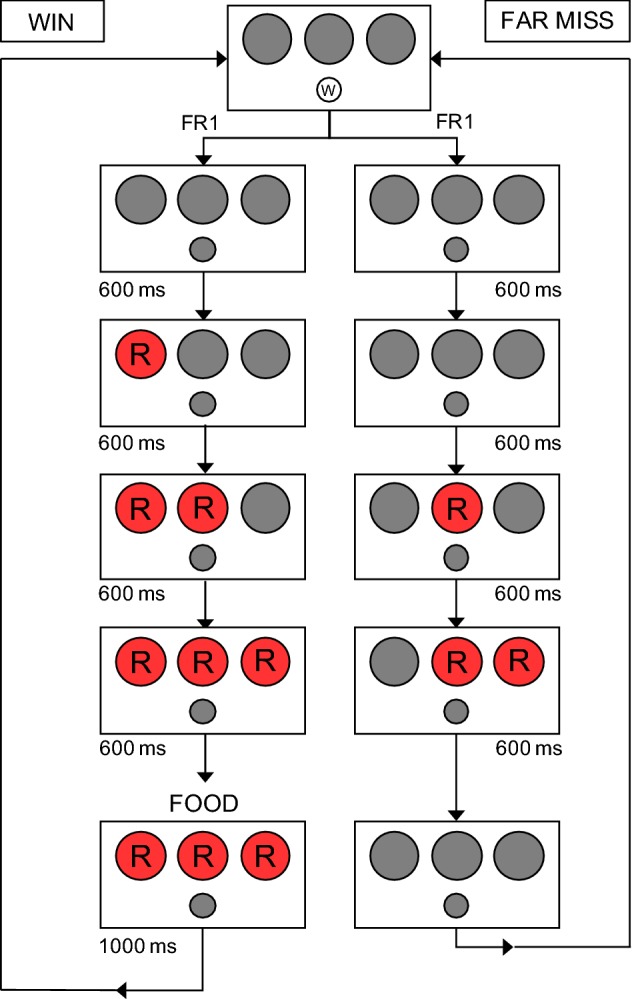
Schematic illustrating the sequence of events for both a win (left column) and a far-miss (right column) during the third component of Experiment 1a’s pre-exposure phase. The letters ‘W’ and ‘R’ indicate the colours white and red respectively. Grey circles indicate empty/unused circles. Circles always illuminate from left to right (Color figure online)

Reels were always presented sequentially from left to right with a 600 ms interval between each presentation. For instance, this was the sequence of events on a ‘winning’ trial: following a peck to the white circle, the white circle disappeared. After 600 ms, the left reel appeared. Then, the middle and right reel each appeared in sequence with a 600 ms interval between each presentation. In total, the sequence was 2.4 s long. Then, the right or left hopper (randomly chosen) rose to provide 1 s access to food pellets (i.e., unconditional reinforcement) with the reels remaining on screen during this period. Finally, the reels reset to black and the white pecking circle returned to occasion the start of the next trial. On all loss trials, each of the intervals still occurred whether or not a red circle appeared so that the total sequence was always 2.4 s long. The reels then reset back to black and the white pecking circle reappeared following the last 600 ms interval. Thus, with the exception of the initial autoshaping phase in this and the subsequent experiments, there were no intertrial intervals following either the win or the loss trials. Figure 1 diagrams this sequence for both win and far-miss outcomes.

#### Ethical Statement

All procedures for the care and use of animals were in accordance with the ethical standards of the University of Alberta, Canadian Council on Animal Care, and were approved by the Bioscience Animal Care and Use Committee (Protocol AUP00002018).

#### Procedure

The experiment was structured as a repeated-measures design with two treatments: the *near*-*miss* and *far*-*miss* treatments. The order of these treatments for each subject was randomly determined according to a Latin square design (i.e., subject × treatment). Each treatment was preceded by a *pre*-*exposure phase* comprised of three components. Only one handler conducted the experimental sessions to further reduce sources of unsystematic variation that can occur in extinction-based designs. Additionally, because the noise of hoppers in adjacent operant chambers might confound the measures taken during periods of extinction, all pigeons had their extinction sessions yoked to occur at the same time and day as other pigeons concurrently running in the experiment.

In the pre-exposure phase, the subjects began on a basic autoshaping procedure (Schwartz and Gamzu 1977), with a fixed-ratio 1 (FR1) contingency built in. Here, a white circle was presented behind the lower response hole for 10 s or until the bird pecked it. After the 10 s or after one peck on the white circle, the circle disappeared and then either the left or right feeding port light illuminated and its hopper raised. When the bird’s head entered the port, it had 1 s to eat from the hopper. Then, the port light extinguished, the hopper lowered, and a 240 s interval began. This process repeated over a 90 min session. All subjects remained on this component for three consecutive sessions.

Following autoshaping, the subjects were put on a FR1 schedule. Responses were made on the lower response hole as before. Following one peck, the white pecking circle disappeared and then the subject had to wait 2400 ms until they were given access to food from either the left or right hopper. The FR1 schedule lasted for one 90 min session. On the next session, the schedule was extended to a FR3 (i.e., three pecks were required to gain access to food). The 2400 ms interval was still implemented after every response. Following one session of FR3, the schedule was extended to a FR6 for one more session. This procedure was followed for all birds with the exception of Bird 76 in Experiment 1b, who displayed unusually low responses after being moved to the FR1 schedule. Autoshaping was reinstated until high rates of responding on the FR1 schedule occurred. Due to the necessity of yoking the extinction sessions, the FR3 and FR6 conditions were skipped in the first half of Bird 76’s sessions to allow for maximal exposure to the contingency in the last pre-exposure component.

In the final pre-exposure component, subjects were put on a random-ratio 5 (RR5) schedule. Similar to a variable-ratio (VR) schedule, a RR schedule typically contains an average response requirement. However, unlike a VR schedule, a RR schedule is determined by pseudo-random number generation as opposed to a predetermined number of reinforced and nonreinforced trials (Hurlburt et al. 1980; Zeiler 1977). In this pre-exposure component, each peck was separated by at least a 2400 ms interval but during this interval, the reel stimuli were now presented at the upper three circles. Birds only gained access to food following wins. All reel outcomes were equiprobable. Specifically, wins, near misses, and all other loss types each occurred 20% of the time and these probabilities were equated every 30 responses. Each bird received fifteen 90-min-long sessions—apart from bird 76 who only received nine sessions of the RR5 schedule in the first half of Experiment 1b.

In the treatment phase, after the pre-exposure phase, all subjects were put on either the near-miss or far-miss treatment. In either treatment, responses on the white key were put on extinction. Specifically, all win reels and unconditional reinforcement were replaced by either the near-miss or far-miss reel patterns. For instance, a subject in the near-miss treatment experienced near misses 40% of the time and no wins, and the proportions of the other loss feedback were left unchanged. The subjects were not presented with any additional cues to signal this change in the schedule. After completing the first treatment phase, all subjects were returned to the pre-exposure phase and then they completed the next treatment condition (i.e., a pigeon that began on the near-miss treatment first completed the far-miss treatment next and vice versa).

### Results and Discussion

Statistical analyses were conducted using R 3.5.0 (R Core Team 2018). The mean difference in resistance to extinction between the near-miss and far-miss treatments was assessed using a paired *t* test on the cumulative number of responses made during extinction. An effect size was calculated using an unbiased estimate of Cohen’s *d* (see equation 11.13 in Cumming 2012). To best account for the reduction in variability offered by paired designs, the *d* estimate was standardized using the standard deviation of the difference scores. To obtain the relative odds in favour of an alternative hypothesis against the null, a JZS Bayes Factor (*BF*_10_) using a medium prior was calculated (Morey and Rouder 2011, 2015).

**Fig. 2:**
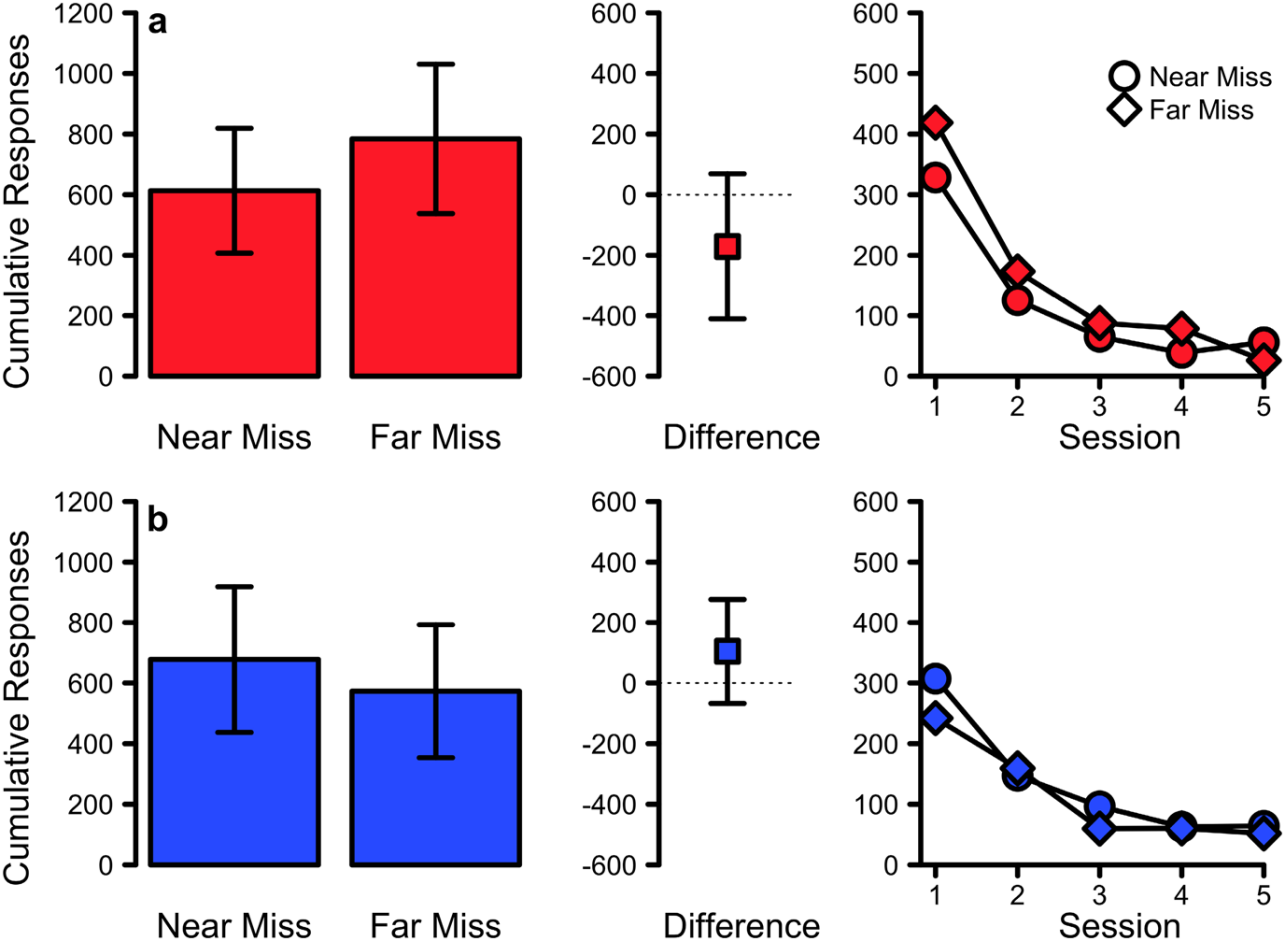
The results of Experiment 1a (top) and 1b (bottom). The left-most plot shows the mean cumulative responses during extinction for the near and far miss treatments. The centre plot shows the mean difference between these treatments. All error bars indicate 95% confidence intervals. The right-most plot shows the mean cumulative responses across the five sessions of extinction testing. Circles and diamonds indicate Near Miss and Far Miss extinction treatments respectively

The results of Experiment 1a are depicted in the top row of Fig. 2. Contrary to the predictions of a near-miss effect, the near-miss treatment showed less overall responding (*M* = 613.25, 95% CI [407.26, 819.24]) in extinction than the corresponding far-miss control treatment (*M* = 784.12, 95% CI [537.73, 1030.52]). However, the difference between the two treatments was not statistically significant, nor was the obtained Bayes Factor meaningfully large, *t*(7) = − 1.68, 95% CI [− 410.75, 69.00], *p* = .136, *d* = 0.53, *BF*_10_ = 0.92.

The results of Experiment 1b are depicted in the bottom row of Fig. 2 and, like experiment 1a, are similarly inconclusive. The mean cumulative responding for the near-miss treatment (*M* = 677.88, 95% CI [436.94, 918.81]) exhibited marginally more responses during extinction than the corresponding far-miss control treatment (*M* = 573.12, 95% CI [353.40, 792.85]), *t*(7) = 1.44, 95% CI [− 67.31, 276.81], *p* = .193, *d* = 0.45, *BF*_10_ = 0.73.

Overall, the results of both Experiment 1a and 1b failed to provide evidence that near misses have a conditionally reinforcing function on the gambling response. However, it may be the case that the supposed near-miss effect is a uniquely human phenomenon and does not apply to non-human or non-primate organisms. While the literature on operant conditioning seems at odds with such a hypothesis, given the strong reliability of reinforcement processes across numerous species, the possibility should nonetheless be considered. The animal-based literature has, in most cases, failed to straightforwardly demonstrate the reinforcing nature of near-miss stimuli whereas the human-based literature has been, at least occasionally, successful. Given this, Experiment 2 adapted and applied Experiment 1a and 1b’s procedure to human participants.

## Experiment 2

### Methods

#### Participants and Apparatus

A total of 296 participants (*Homo sapiens*) were recruited from the research participant pool of introductory psychology courses at the University of Alberta. The sample consisted of 192 females and 104 males, with a mean age of 19.3 years. Of these, N = 178 were used in the final analyses (see results section for details on exclusion criteria). A projected sample size of at least 128 subjects, post exclusion criteria, was determined using the G*power 3.1. computer program (Faul et al. 2007) for a “medium” sized effect (*f* = .25) at an 80% power level for detection of main effects and interactions. Participants were incentivized with partial course credit and a small monetary bonus of up to $9.50 CAD that depended on their final earnings. Sessions were conducted in a space containing fifteen individual testing rooms located around the perimeter. The task was completed on a computer with a mouse and keyboard. E-Prime^®^ 2.0 Professional software was used to present stimuli and record responses.

This experiment was adapted from Experiments 1a and 1b. The stimuli were presented on a black background, and three $$2{\raise0.5ex\hbox{$\scriptstyle 1$} \kern-0.1em/\kern-0.15em \lower0.25ex\hbox{$\scriptstyle 8$}}$$ in. circles that comprised the reel patterns were shown at the top of the screen. Their locations were outlined by a grey border. Reel patterns were presented in the same manner as Experiments 1a and 1b. A 1½ in. white circle located just below the middle reel stimulus location served as the response key. The response key was also outlined with a grey border, and text within it read “Click Me!”.

#### Ethical Statement

All procedures performed in studies involving human participants were approved by the University of Alberta Research Ethics Board (Protocol Pro00058367) and were in accordance with the Tri-Council Policy Statement on the Ethical Conduct of Research Involving Humans.

#### Procedure

Participants read an information and consent form. After signing a form to provide their consent for participation. They were assigned to individual testing rooms and instructed to begin. After being prompted to enter their age and sex on the computer, a brief set of instructions was presented to them on screen for a minimum of 1 min. Instructions told participants to try and earn as much money as possible. It also informed them that they could stop playing at any time to collect their money.

Participants were assigned randomly to one of two groups and then further randomly assigned to one of two treatments. Groups were analogous to Experiments 1a and 1b: participants in the *S*^*D*^ group only saw red circles in the reel patterns, and participants in the *S*^*D*^*&S*^*∆*^ group saw both red and blue circles but only red circles signalled wins. The treatments were also similar to Experiments 1a and 1b: near misses or far misses replaced the wins during extinction.

Experiment 2’s pre-exposure phase was similar to the third component of the pre-exposure phases from Experiment 1. The first 15 trials were structured such that the first, fifth, and eleventh trials were wins. This was to prevent participants from encountering an early string of losses that could possibly occur due to the RR schedule and produce a negative monetary score. The remaining 285 trials proceeded according to a RR5 schedule, with the probabilities of all outcomes being equated every 15 trials. Participants began with $0.50 and their score was always shown at the bottom of the screen. It cost participants $0.05 for each play on the response key and this was immediately deducted from their score. Wins awarded $0.40 and they were signalled by the appearance of “Win! +40¢” in large bright yellow text beneath the response key.

The treatment phase occurred after 300 trials, at which point plays on the response key were put on extinction: clicking it still cost $0.05 but doing so no longer produced wins. Participants in the near-miss treatment had the wins replaced by near misses and participants in the far-miss treatment had wins replaced by far misses. Unlike some other extinction-based studies (Kassinove and Schare 2001; MacLin et al. 2007) participants were not presented with any cues to signal this change nor were they ever told that such a change would occur. Participants could continue playing until 50 min had elapsed. When participants were ready to leave, they were paid according to their final score and given a debriefing form about the experiment.

### Results and Discussion

Statistical analyses were conducted only on those participants for whom the task was observed to be sufficiently reinforcing. This was defined as completing more than 300 trials. This criterion was set because 300 was the minimum number of trials a person would have to complete before entering the extinction phase. Of the total sample, 178 (60%) continued through to the extinction phase. Within the *S*^*D*^ group (n = 91), there were 28 females and 16 males assigned to the Near Miss treatment and 36 females and 11 males assigned to the Far Miss. Within the *S*^*D*^*&S*^*∆*^ group (n = 87), there were 26 females and 19 males assigned to the Near Miss treatment and 30 females and 12 males in the Far Miss. The remaining, 118 (40%), participants did not make it to the extinction phase of the experiment. Of these, 48% were in the S^D^ group and 52% were in the S^D^&S^∆^ group. As in Experiment 1, analyses were conducted on the cumulative number of responses made during extinction using R 3.5.0 (R Core Team 2018). Because statistical and visual inspection of the data revealed heavy positive skewness across all groups and treatments, *G*_1_ = 2.59 (for details on the *G*_1_ skewness measure, see Joanes and Gill 1998), a base-10 logarithmic transformation was conducted to make the data symmetrical, *G*_1_ = 0.82, and permit parametric analyses using a 2 × 2 Factorial ANOVA. Effect size estimates in terms of Cohen’s F (*f*) were obtained for each main effect and interaction term. A corresponding JZS Bayes Factor showing the relative odds of each term against the model’s intercept is also reported (Morey and Rouder 2011, 2015).

**Fig. 3:**
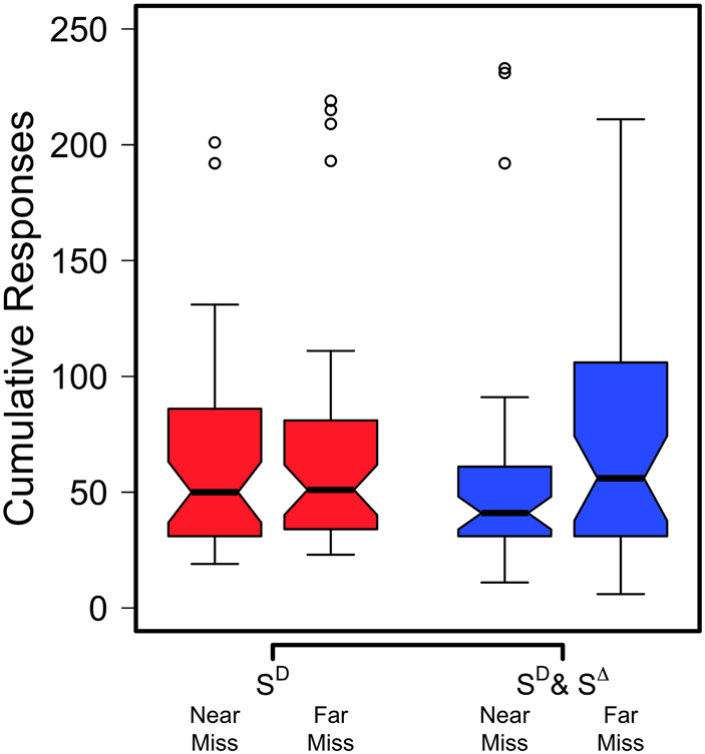
The results of Experiment 2. Plot displays notched box-and-whisker plots diagramming cumulative responses during extinction for S^D^ and S^D^&S^∆^ groups across the near-miss and far-miss treatments. Whiskers follow the 1.5 × IQR rule. Hollow circles show data points falling outside the whisker edges

Figure 3 shows the cumulative number of responses made for each group in Experiment 2 plotted in untransformed units using notched box-and-whisker plots. There was a non-significant main effect of both miss type (near miss vs. far miss), *F*(1, 174) = 2.12, *p* = .148, *f* = 0.11, *BF*_10_ = 0.42, and stimulus group (S^D^ vs. S^D^&S^∆^), *F*(1, 174) = 0.01, *p* = .92, *f* = 0.01, *BF*_10_ = 0.16. A non-significant interaction between miss type and stimulus group was also obtained, *F*(1, 174) = 1.49, *p* = .22, *f* = 0.09, *BF*_10_ = 0.03.

The results of Experiment 2 corroborate the findings of Experiment 1 as well as other extinction studies that failed to find a reinforcing effect of near-miss stimuli on human participants. Granted, Experiment 1 and 2 have only shown an *absence of evidence* for a conditional reinforcement effect. However, if it can be shown that an effect analogous to the near-miss effect in extinction could not be obtained despite the presence of a probable conditional reinforcer, then that would be indicative of *evidence of absence* for enhanced persistence of conditional reinforcers in extinction. Experiment 3 took advantage of the fact that discriminative stimuli (i.e., stimuli which occasion the occurrence of a consequence for a behaviour) will frequently also acquire respondent conditioned stimulus (CS+) functions due to their contingent relationship with unconditional reinforcers (i.e., unconditional stimuli). For instance, food predictive stimuli will often elicit consummatory responses in animals. In the case of pigeons, this usually takes the form of pecking at the food predictive stimulus with an open beak (Schwartz and Gamzu 1977). Assuming that respondent conditioning is the processes by which neutral stimuli acquire their conditionally reinforcing function (which remains the most dominant well-supported view and forms the basis for the logic of extinction-based tests of persistence; Williams 1994), then evidence of a conditioned response occurring on the putative conditional reinforcer should be indicative of its potential to reinforce in extinction. If this is not observed, then one could argue that extinction-based tests—of the kind used in prominent near-miss studies (Cote et al. 2003; Kassinove and Schare 2001)—are unreliable and perhaps a poor metric of the near miss’s effect on gambling behaviour. Therefore, Experiment 3 simplified the reel stimuli by arranging the pre-exposure phase contingencies so that the respondent CS+ function of the feedback stimuli could be verified independently of the extinction procedure. Following this, the effect of those stimuli were tested to see if they could be used to increase persistence during extinction.

## Experiment 3

### Methods

#### Subjects and Apparatus

An additional 8 homing pigeons that were not used in Experiments 1a and 1b were randomly selected. The apparatus was the same as those used in Experiments 1a and 1b.

#### Ethical Statement

All procedures for the care and use of animals were in accordance with the ethical standards of the University of Alberta, Canadian Council on Animal Care, and were approved by the Bioscience Animal Care and Use Committee (Protocol AUP00002018).

#### Procedure

Like Experiment 1, a repeated-measures design with two treatments was used and the order the treatments was randomly determined by a Latin square (i.e., subject × treatment). Subjects began on a three-component pre-exposure phase followed by a treatment phase. In all phases, the sessions lasted for 90 min or 200 food reinforcements, whichever occurred first. If a bird obtained all 200 reinforcements before the 90 min had elapsed, it remained in the box with an inoperative blank screen. Only one handler conducted the experimental sessions during the treatment phase to reduce unsystematic variation.

The first component of the pre-exposure phase was the same as the autoshaping procedure from Experiment 1a. In the next component, the pigeons were put on a FR1 schedule for one session that reinforced responses to the white pecking circle at the lower hole in the barrier. After a single response the circle turned black and was followed by a 2400 ms interval before reinforcement was delivered. In the third component, the pigeons were moved to a pure RR2 schedule with the number of pecks determined randomly according to a 0.5 probability of any given response leading to food reinforcement. After completion of the RR2 schedule, a single S^D^ or S^∆^ feedback stimulus (indicating reinforcement and nonreinforcement respectively) was displayed for 2400 ms at the center hole of the upper three circles in the plastic barrier. Following S^D^ presentations, the bird was given 1 s to access food, during which the S^D^ remained present (delayed conditioning). The S^D^ then disappeared concurrently with reappearance of the white response circle. Non-reinforced trials displaying the S^∆^ were followed immediately by the termination of S^∆^ and reappearance of the white response circle.

The S^D^ and S^∆^ stimuli could consist of either a yellow circle with a thin black horizontal line intersecting it or a light blue circle with a thin black vertical line intersecting it. Half the birds were randomly selected to receive a yellow S^D^ and blue S^∆^ while the other half received a blue S^D^ and yellow S^∆^. To assess conditional responding elicited by the S^D^, pecking responses made to the S^D^ and S^∆^ stimuli were recorded at the millisecond interval.

Following five sessions on the RR2 schedule, subjects were put on a treatment phase for three sessions where responses to the white pecking circle were put on extinction. Unconditional reinforcement was removed and the proportions of feedback were modified. In the *S*^*D*^*80%* treatment, subjects received the S^D^ feedback stimulus 80% of the time and the S^∆^ feedback stimulus 20% of the time. Subjects in the *S*^*D*^ 20% treatment received the S^D^ feedback stimulus 20% of the time and the S^∆^ feedback stimulus 80% of the time. After the first extinction phase, subjects returned to the pre-exposure phase and then they completed the other treatment condition.

### Results and Discussion

To evaluate the conditional responding occurring on the designated S^D^ stimulus, a discrimination index (*I*) for each pigeon was calculated using the equation *I* = *S*^*D*^/(*S*^*D*^ + *S*^*∆*^) where *S*^*D*^ and *S*^*∆*^ represent the cumulative amount of responses made respectively during each stimulus across all pre-exposure phase sessions. This was tested against a prediction of no discrimination (*I* = 0.5). The data were collapsed across both blocks of pre-training since there was no reason to expect an effect of order. The index means across the first and second blocks of pre-exposure corroborate this supposition. The first block obtained a mean discrimination index of *M* = 0.985, 95% CI [0.973, 0.997] and the second of *M* = 0.998, 95% CI [0.996, 1.001] respectively. Across all pre-exposure sessions, an extreme effect on responding to the S^D^ relative to the S^∆^ was observed, *t*(7) = 187.73, *p* < 0.001, 95% CI [0.986, .999], *d* = 118.90, *BF*_10_ > 150). This indicates that the presence of the S^D^ relative to the S^∆^ had a strong CS+ function on pecking, a robust and critical feature of conditionally reinforcing stimuli generally (Gollub 1977). This occurred despite the fact that pigeons were never trained to respond to this location, had no prior experience with these stimuli, and that responses made to this stimulus had no impact on the sequence or timing of events.

**Fig. 4:**
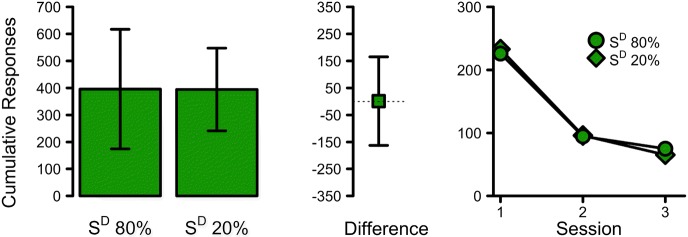
The results of Experiment 3. Left-most plot shows the mean cumulative responses during extinction for the S^D^ 80% and S^D^ 20% treatment. The centre plot shows the mean difference between these treatments. All error bars indicate 95% confidence intervals. The right-most plot shows the mean cumulative responses across the three sessions of extinction testing. Circles and diamonds indicate the S^D^ 80% and S^D^ 20% extinction treatments respectively

Statistical analyses on cumulative number of responses made during extinction were calculated as per Experiment 1. The results (Fig. 4) showed a non-significant effect and no support of a difference between the tested extinction treatments, *t*(7) = 0.02, *p* = 0.985, 95% CI [− 162.38, 165.13], *d* = 0.01, *BF*_10_ = 0.34, despite the strong evidence that the S^D^ feedback stimulus functioned as a conditional stimulus during the pre-exposure phase. This strongly implies that whatever reinforcing function the reels on a slot machine may have, it is likely independent of and thus does not reliably reinforce prolonged play during a period of extinction (i.e., a period of consistent losses).

## General Discussion

At present, some of the most prominent lines of experimental evidence in favour of the near-miss effect come from a select set of studies employing extinction-based persistence tasks (Cote et al. 2003; Kassinove and Schare 2001). The present studies failed to replicate positive evidence of a near-miss effect on gambling persistence and instead corroborated other less-cited experimental studies that have not demonstrated a reliable reinforcing effect of near-miss stimuli on extinction-based, choice-based, or observing-response-based tasks (Fortes et al. 2017; Ghezzi et al. 2006; MacLin et al. 2007; Peters et al. 2010; Reid 1986; Stagner et al. 2015; Strickland and Grote 1967; Witts et al. 2015). More notably, Experiment 3 of the present study demonstrated that the use of an extinction-based treatment in evaluating the function of a putative conditional reinforcer is, at best, unreliable and, at worst, ineffective. This raises a salient point about the nature of reinforcing effects: even if it is assumed that slot machine contingencies produce some type of conditionally reinforcing function on the reels, there is no guarantee that the generated function will act as reinforcement for the frequency of the gambling response itself. Specifically, there is no guarantee that it will reinforce gambling persistence. Instead, the reinforcement generated on the reels may only serve to reinforce other types of responses such as initial machine selection or amount bet; or perhaps near misses may only serve a respondent function whereby various conditional emotional responses are elicited (Clark et al. 2012; Dixon et al. 2013).

A possible limitation of the present work is that the contingencies in effect across all three experiments’ pre-exposure phases only ever produced a net gain—a fact uncharacteristic of slot machines and casino games in general. However, this was done to maximize the possibility of generating a conditionally reinforcing effect of near misses. Even despite this net gain, around 40% of the participants in Experiment 2 opted to cash out of the experiment prior to extinction occurring, and sometimes well before the maximum payout was reached. Although this seems like a large proportion, it is worth pointing out that similar studies (e.g., Kassinove and Schare 2001) have required a requisite number of trials to occur before participants were permitted to cash out, which complicates assessment of the task’s reinforcing value. In contrast, the present study allowed participants to cash out at any point. Because of these differences, we cannot definitively say that a 40% attrition rate is high or low for these types of studies, but this may be indicative of a problem with studying undergraduate populations for whom money may be only weakly reinforcing at these levels.

A second potential limitation is that participants were not required to risk losing their own money to play, as is the case with real-world slot machine gambling. For ethical reasons, we could not require participants to ante up with their own money, but they did risk losing money already earned by continuing to play during extinction. Indeed, many participants in both the near-miss and far-miss conditions continued to play for more than 100 trials after the extinction phase was initiated—which is consistent with the increased persistence seen following exposure to intermittent reinforcement schedules known as the partial-reinforcement effect (Nevin 1988)—but this resulted in a loss of the money previously earned. Moreover, continuing to play involved an investment of time and energy.

A third possible limitation concerns the simplistic nature of the stimuli used. Slot machines generally incorporate reel spinning and all manner of varied stimuli, some of which are quite complex. The present set of experiments employed only very basic shapes, patterns, and outcomes. However, this too was by design: if near misses do produce a meaningful effect on gambling persistence, then the effect should be independent of conventional gambling stimuli, which are varied and largely arbitrary. In an effort to reduce unsystematic variation, the present studies used basic neutral stimuli and patterns that would limit pre-existing biases and make learning of the contingencies more straightforward.

A fourth limitation is that certain boundary conditions might be necessary to generate the near-miss effect. That is, our studies may not have included the specific set of variables needed to produce the near-miss effect. While we do not discount this possibility, the strongest positive evidence for near-miss effects on gambling persistence (Cote et al. 2003; Kassinove and Schare 2001) does not make explicit any such boundary conditions. Consequently, there was no a priori reason to believe that anything but near misses ought to be manipulated to see a resulting effect on behaviour. Moreover, from our examination of the literature, we were unable to determine any conditions that consistently predicted whether a near-miss effect would emerge.

One potential boundary condition could be the population of participants. For example, it could be that near-miss stimuli only enhance persistence in problem gamblers, and not in non-gamblers. Some studies, for example, have found that near-miss stimuli have produced physiological or neural responses to near-miss stimuli in problem gamblers but not in non-problem gamblers (Dymond et al. 2014; Habib and Dixon 2010). However, these studies did not show a behavioral effect on gambling persistence and it is impossible to know whether the reaction to near-miss stimuli was a contributor to, or a consequence of the gambling problem. Moreover, the most frequently cited positive evidence for near-miss effects has come from studies of non-problem gamblers. Thus, a challenge for future research would be to show that near-miss events with initially neutral stimuli (i.e., not already conditioned by prior gambling experience) produce behavioral persistence in problem gamblers.

Finally, there could be factors that are external to the experiment that could have undermined the extinction procedure—especially for humans. Witts et al. (2015) commented that washroom needs or simply having access to more reinforcing activities outside of the experiment can create competing contingencies that influence participants to leave. The results from Experiment 2 support this: the proportion of participants that cashed out before extinction (and thus before earning the maximum reward) suggests that the amount of money that they were earning was not sufficiently reinforcing to keep many engaged in the task. Providing a larger monetary incentive could potentially alleviate this, but how much more would be needed is unknown. This illustrates one of the advantages of using animal models: since food is a powerful reinforcer, greater control can be obtained over the subject’s behaviour. Further, motivating operations can be adjusted and other extraneous variables are often more easily controlled (e.g., learning histories).

Near-miss research has gained much attention in recent years. Most of this research, however, has examined measures other than behavioral persistence, such as physiological reactions, response latencies, or data from questionnaires. These effects are presumed to be consistent with prolonging slot machine play, but few studies have demonstrated a reinforcing effect on persistence explicitly. If near misses are truly prolonging gambling behaviour, then why are there not numerous experimental studies that have conclusively and consistently demonstrated this effect? It is profoundly telling that decades after B.F. Skinner first acknowledged the possibility that near misses might be reinforcing gambling responses, there is still no experimental paradigm that seems able to consistently demonstrate this effect on gambling behaviour.

Given that the near-miss effect on gambling persistence was founded on an early and imprecise account of conditional reinforcement (Fantino 1977; Skinner 1953), near-miss research may have been misguided from the start. Furthermore, some studies—including the present work—seem to do more to challenge the belief that near misses prolong gambling. If near misses do lead to prolonged gambling, the effect appears to be limited or idiosyncratic (Witts et al. 2015). Nevertheless, 66 years after B.F. Skinner first proposed the idea, adherence to the belief that near-miss outcomes reinforce gambling persistence has remained strong. Our research questions the underlying premise that conditional reinforcement by near-miss stimuli should increase persistence of gambling behavior during extinction.
